# Diagnostic value of endoscopic ultrasound elastography for benign and malignant digestive system tumors

**DOI:** 10.12669/pjms.35.5.1075

**Published:** 2019

**Authors:** Hongna Lv, Guangchao Zhu, Long’an Zhou

**Affiliations:** 1 Hongna Lv, Department of GI Medicine, Binzhou People’s Hospital, Binzhou, 256600, China; 2 Guangchao Zhu, Department of Internal Medicine, Wudi People’s Hospital, Wudi, 251900, China. Binzhou People’s Hospital, Binzhou, 256600, China; 3 Long’an Zhou, Department of Gastrointestinal Surgery, China

**Keywords:** Benign and malignant, Digestive system tumors, Endoscopic ultrasound real-time elastography, Strain ratio

## Abstract

**Objective:**

To investigate the effect of endoscopic ultrasound real-time tissue elastography in differential diagnosis of benign and malignant digestive system tumors.

**Methods:**

Forty-two patients with solid tumors of digestive system who were admitted to our hospital between October 2017 and October 2018 were selected. All patients were diagnosed by endoscopic ultrasound real-time tissue elastography. Elastography score was used. The strain ratios (SR) of the lesion and the surrounding control tissues were measured and compared.

**Results:**

Lesions with elastography score no more than two points were evaluated as benign, while lesions with elastography score no less than three points were evaluated as malignant. The difference of the elastography score between the benign lesion group and malignant lesion group was statistically significant (P<0.05). The sensitivity, specificity and accuracy of endoscopic ultrasound elastography in the diagnosis of malignant tumors of digestive system were 91.4%, 88.9% and 87.5%, respectively. The SR of the benign lesions ranged from 0.01 to 7.34, with a median SR of 7.33; the SR of the malignant lesions ranged from 1.01 to 47.66, with a median SR of 20.07. The SR of the benign lesions was significantly lower than that of the malignant lesions (P<0.05).

**Conclusion:**

Elastography of benign and malignant tissues of digestive tract tumors has different image characteristics. Endoscopic ultrasound real-time tissue elastography is effective in differential diagnosis of digestive tract tumors as it can effectively determine whether a tumor is benign or malignant and improve diagnostic accuracy.

## INTRODUCTION

Ultrasound elastography is a new diagnostic technique developed in recent years. It can be used to differentiate benign and malignant tumors of superficial organs such as breast, thyroid and lymph nodes.^1^-^3^ However, there are some limitations in the study of deep organs. Abdominal ultrasound is difficult or impossible to obtain satisfactory elastography images of pancreas, liver and gastrointestinal submucosal lesions. Endoscopic ultrasonography (EUS) can detect intraluminal tumors with ultrasound probe. Featured by short detection distance, high resolution to tissues and small interference, EUS breaks the limitation of ultrasound in the diagnosis of some diseases of the digestive system.^4^,^5^ EUS not only can clearly show the small tumors of upper digestive tract, but also can accurately judge the size and local infiltration range of lymph nodes, mediastinum, pancreas, submucosal lesions.

However, EUS B-mode gray-scale imaging is difficult to distinguish between benign and malignant tumors, which limit its diagnostic value.^6^ EUS real-time elastography is a new imaging technology, which can visualize and quantify tissue elasticity in EUS. It effectively complements the shortcomings of EUS in identifying the nature of lesions and differentiating benign from malignant tumors.^7^,^8^ At present, endoscopic elastography is still in its infancy in China, so there are few reports on the application of endoscopic elastography in the evaluation of benign and malignant digestive system tumors. The purpose of this study was to further demonstrate the value of endoscopic elastography score and strain ratio (SR) in differential diagnosis by analyzing relationships of endoscopic elastography score and SR with benign and malignant tumors in the digestive system.

## METHODS

### General data

In this study, 42 patients with digestive system tumors who were admitted to our hospital from October 2017 and October 2018 were selected. There were 63 solid tumors in the digestive system. All patients were diagnosed as digestive tract tumors by B-mode ultrasound, computed tomography (CT) and endoscopy. There were 25 males and 17 females, and they aged 35-68 years, with an average age of (51.35±13.51) years. There were 18 benign lesions and 45 malignant lesions. There were 12 cases of pancreatic cancer, 9 cases of hepatocellular carcinoma, 7 cases of hepatic hemangioma, 3 cases of hepatic abscess, 7 cases of gastric stromal tumors and 4 cases of esophageal leiomyoma. The study protocol has been approved by the ethics committee of our hospital, and all the research subjects have signed informed consent.

### Diagnostic method

Hitachi EUB-8500 color Doppler ultrasound diagnostic instrument and PENTAX EG-3270 UK fan scanning electronic endoscopy were used. First, B-mode gray-scale imaging was used for conventional probe. After grasping the location, size and echo characteristics of the lesions, ultrasound elastography was performed on the target area. The real-time elastography mode was set, and the region of interest of ultrasound was adjusted to the appropriate size. The elastography images of the target area were obtained through breathing movement, pulsation of thoracoabdominal artery and pressure of probe.

After the operation, the elastography images and SR were analyzed and the elastography score was given.

### Evaluating indicators

Scoring criteria of EUS elastography were as follows. The hardness of lesions was determined according to the color of real-time tissue elastography images, blue for hard, red for soft and green and yellow for the hardness between soft and hard. Elastography was scored using five-point elasticity scoring system.^9^ One point was given when the lesion and surrounding tissues was completely covered with green; two points was given when the lesion area was mixed with blue and green, mainly with green; three points was given when the lesion area was mainly blue, with green in the surrounding area; four points was given when the lesion area was completely covered with blue; five points was given when the lesion area was totally blue and the surrounding tissues showed blue. Malignant lesions scored more than three points, while benign lesions scored less than two points. Elastic SR analysis method was used. The lesion area was regarded as the region of interest A,^10^ then the surrounding tissue at the same level was regarded as the region of interest B, the control, and SR was calculated using the formula: SR = SR of B/SR of A.

### Statistical analysis

SPSS18.0 was used for statistical analysis of data. The comparison of SR between benign and malignant groups was performed by Mann-Whimey U test, and the test level was a=0.05. P<0.05 meant significant difference.

## RESULTS

### Analysis of EUS elastography score

**Fig.1:**
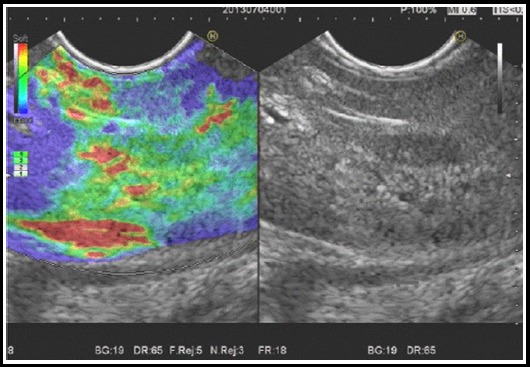
Hepatic hemangioma with 2 points of elastography score.

**Fig.2:**
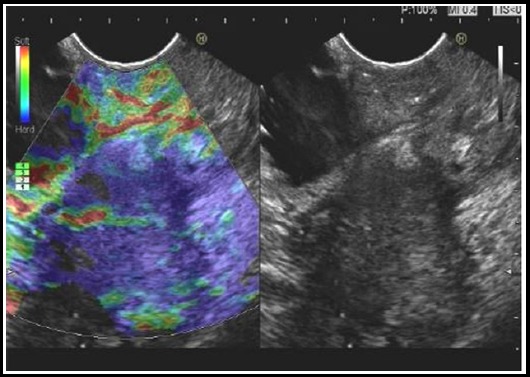
Pancreatic cancer with 5 points of elastography score

Three out of 18 benign lesions were scored for 3 points and misdiagnosed as malignant, and the remaining 15 lesions were scored 2 points or less. The elastography image of a typical case of benign lesion is shown in Figure 1. Of 45 malignant lesions, 2 lesions were misdiagnosed as benign, and the remaining 43 were scored 3 points or more. The elastography image of a typical case of malignant lesion is shown in Figure 2. The lesions with no less than 3 points were evaluated as malignant lesions and those with no more than 2 points were evaluated as benign lesions. Two groups, the benign lesion group and malignant lesion group, were established. There was a significant difference in the elastography score between the two groups (P<0.05, Table-I).

**Table I:** Elastography scores of benign and malignant lesions.

Pathological type	Number of lesions/n	Elastography score				

		1 point	2 points	3 points	4 points	5 points
Malignant	45	0	2	9	20	14
Benign	18	6	9	3	0	0

### Effectiveness of EUS elastography in Diagnosis

The diagnostic accuracy, sensitivity and specificity of EUS real-time tissue elastography were 92.06% (58/63), 95.56% (43/45) and 83.33% (15/18), respectively (Table-II).

**Table II:** EUS elastography and pathological results.

Elastography	Pathological results		Total

	Benign	Malignant	
Benign	15	2	17
Malignant	3	43	46
Total	18	45	63

### SR comparison between benign and malignant lesions

The SR of the lesions was calculated according to the SR analysis method aforementioned, and the benign and malignant lesions were compared between groups. There were 18 benign lesions, with SR ranged from 0.01 to 7.34 (median 7.33) and 45 malignant lesions, with SR ranged from 1.01 to 47.66 (median 20.07). The SR of the benign lesion group was significantly lower than that of malignant lesion group (P<0.05, Table-III).

**Table III:** Elastic SR between benign and malignant lesions (%).

Pathological type	Number of lesions/n	Range of elastic SR	Median elastic SR
Benign	18	0.01~7.34	7.33
Malignant	45	1.01~47.66	20.07

## DISCUSSION

EUS real-time tissue elastography is a new endoscopic diagnostic technique which combines the techniques of ultrasound elastography and ultrasound endoscopy. It can compress the target area with the help of breathing, pulse and moving ultrasound probe, so as to measure the acoustic parameters of the region of interest in the image. Moreover, it can display the elasticity coefficient of tissues through different colors. It provides a new way for the differentiation of benign and malignant tumors.^11^,^12^ As the elasticity coefficients of tumors, inflammatory tissues and normal tissues are different, malignant tumors are more rigid and less compliant, and the elasticity coefficients of normal tissues or benign lesions are much smaller than that of malignant tumors, ultrasound elastography can differentiate benign from malignant lesions by evaluating the hardness of tissues of different lesions.^13^,^14^

At present, the five-point scoring system is the main method in the diagnosis of benign and malignant lesions; a higher score is corresponding to a higher elasticity coefficient of a lesion compared to normal tissues.^15^ In this study, the lesions with no less than 3 points were evaluated as malignant lesions and those with no more than 2 points were evaluated as benign lesions. The results showed that there was a significant difference in the elastography score between benign lesions and malignant lesions (P<0.05), indicating that the tissue hardness of malignant lesions was greater. The main reason was that the compliance and hardness of malignant tumors were lower than that of benign tumors.^16^

In addition, the results showed that the diagnostic accuracy, sensitivity and specificity of EUS real-time tissue elastography were high, 92.06%, 95.56% and 83.33% respectively, which indicated that EUS elastography had a high diagnostic effect. Giovannini et al. performed EUS-RTEI on 121 patients with space occupying pancreatic lesions^17^, took 1~1 points and 3~5 points as the elasticity diagnostic criteria of benign and malignant lesions, and found that the sensitivity and specificity of EUS-RTEI in the diagnosis of malignant pancreatic space-occupying lesions were 92.3%, 80.0% and 89.2% respectively, which were similar to the results of this study. However, the quality of elastography depends on the quality of data acquisition, how to carry out fast and effective data acquisition, how to set the best elastic parameter classification, and how to distinguish and reduce artifacts, etc., all of which are keys determining the clinical practicability and accuracy of elastography and also the matters needing attention in the operation and analysis of images.

SR refers to the ratio of B/A (lesion area as A and the surrounding soft tissue at the same lesion as the control, B). SR converts the color distribution of the elastography image of lesions and surrounding tissues into numerical values, objectively reflects the hardness of lesions, and avoids the influence of some subjective factors of ultrasound elastography, which is a quantitative indicator in the diagnosis of ultrasound elastography that can help accurately determine the benign and malignant tumors.^18^-^20^ The results of the present study showed that the SR of benign lesions ranged from 0.01 to 7.34 (median 7.33) and that of malignant lesions ranged from 1.01 to 47.66 (median 20.07) and the SR of malignant lesions was significantly higher than that of benign lesions, which was similar to the research result of Itokawa et al.^21^ It indicated that the elastic SR of the benign lesion group was lower, which was because that benign lesions were soft and had large deformation and malignant lesions were hard and had small deformation.

## CONCLUSION

To sum up, this study preliminarily showed that EUS real-time tissue elastography was effective in differential diagnosis of digestive system tumors and could effectively determine the benign and malignant tumors and improve the accuracy of diagnosis. The operation of EUS real-time tissue elastography is simple and the result can be reexamined. It is basically non-invasive to human body in tolerable conditions. It is of great value in the diagnosis and treatment of digestive system tumors. However, EUS-RTEI is only an auxiliary examination method which cannot completely replace the puncture technique. Elastography is an important supplementary examination method when patients cannot tolerate EUS-fine needle aspiration (FNA).

### Authors’ Contribution

**HNL, GCZ & LAZ:** Study design, data collection and analysis.

**HNL&GCZ:** Manuscript preparation, drafting and revising.

**HNL:** Review and final approval of manuscript.
